# The Double-Edged Sword of a Calling: The Mediating Role of Harmonious and Obsessive Passions in the Relationship between a Calling, Workaholism, and Work Engagement

**DOI:** 10.3390/ijerph17186724

**Published:** 2020-09-15

**Authors:** Jinsoo Choi, Surawut Permpongaree, Nanhee Kim, Yeeun Choi, Young Woo Sohn

**Affiliations:** 1 Psychology Department, Yonsei University, 50 Yonsei-ro, Seodaemun-gu, Seoul 06695, Korea; harveychoi93@yonsei.ac.kr (J.C.); surawut.p@hotmail.com (S.P.); sksgml7025@yonsei.ac.kr (N.K.); 2 Department of Psychology, University of Central Florida, 4111 Pictor Lane, Orlando, FL 32816, USA; ychoi@knights.ucf.edu

**Keywords:** calling, harmonious passion, obsessive passion, work engagement, workaholism

## Abstract

Even though research on perceiving a calling has been growing, our understanding of its double-edged sword effects and psychological mechanisms remain unclear, especially in terms of work engagement and workaholism. Based on the heavy working investment (HWI) and dualistic model of passion (DMP) theories, we established a dual-path structural model to examine the effects of callings on work engagement and workaholism through two types of passion: harmonious (HP) and obsessive (OP) passions. Our results showed that the association between perceiving a calling and work engagement was partially mediated by HP, while the association between perceiving a calling and workaholism was fully mediated by OP. This study contributes to the literature in that it reveals how perceiving a calling has different effects on work engagement and workaholism through the HWI theoretical lens, as well as the mediating roles of HP and OP, based on the DMP theory. Our findings can be practically applied in organizations and counseling.

## 1. Introduction

A calling is an attitude or cognition process that allows individuals to experience positive meaning and fulfillment in their work, benefitting both the individual and the organization [1,2,3]. For example, workers with a calling tend to experience positive and fulfilling work-related participation characterized by vigor, dedication, and absorption, namely, work engagement [4]. However, recently, it has been suggested that perceiving a calling might be a double-edged sword for workers, leading to negative work-related outcomes as well as positive ones. Specifically, perceiving a calling was found to increase workaholic behaviors such as working excessively and compulsively [5].

Several studies have investigated the positive effects of perceiving a calling on workaholism, but there have been mixed results: some reported a significant relationship between the two variables, while others did not [5,6,7]. We assumed that such mixed results were due to a lack of research on the psychological mechanisms underlying the relationship between perceiving a calling and workaholism. Thus, this study aims to clarify these mixed results by investigating the association and respective mechanisms of perceiving a calling and workaholism.

Previous research has revealed that perceiving a calling is positively associated not only with work engagement but also with workaholic behaviors. However, the question “how is perceiving a calling positively associated with both work engagement and workaholism?” remains unanswered. In this study, we attempt to answer this research question based on the heavy work investment (HWI) concept: the high investment of both time and effort at work [8]. In this regard, workaholism and work engagement can be understood through one unified theoretical lens; in other words, as a negative or positive type of HWI [9,10]. Moreover, a recent piece of meta-analysis research showed that workaholism and work engagement are empirically distinct but are somewhat overlapping in psychological concepts [11]. We expect that perceiving a calling would be positively related to HWI since workers with a calling tend to love their work and regard it as an important part of their identity [12], being more likely to invest time and effort at work [2,3]. Thus, we posit that the workplace behaviors of workers with a calling can be manifested as a form of heavy working investment: work engagement and workaholism.

To determine which factors differentiate workaholic behaviors from work engagement in workers who have a calling, we utilized the dualistic model of passion (DMP) [13], according to which two types of passion exist: harmonious and obsessive passions. Harmonious passion (HP) is an autonomous internalization that leads individuals to choose to engage in activities that they like. On the other hand, obsessive passion (OP) is a controlled internalization of activities in one’s identity that creates an internal pressure to engage in activities that the person likes. Previous research has shown that perceiving a calling is closely related to passion [14]; in turn, passion is also related to HWI [8]. In this regard, we posit that passion (i.e., HP and OP) can mediate the relationship between perceiving a calling and work engagement or workaholism.

Taken together, this study aims to utilize a dual-path mediation model to investigate the positive relationship between perceiving a calling and the types of HWI (i.e., workaholism and work engagement), as well as the mediating roles of HP and OP in the aforementioned relationship. We expect that this study will contribute to the literature by revealing the double-edged sword effects of perceiving a calling on work engagement and workaholism and the psychological mechanisms underlying these relationships based on the dualistic model of passion.

### 1.1. HWI (Workaholism and Work Engagement)

Work engagement and workaholism are two different but similar psychological concepts: they both refer to working hard in terms of behavior, but each concept has its own distinctive features in terms of cognition. Since Oates first suggested the concept of workaholism to describe people who are addicted to work [15], it has been defined in various ways by many scholars. For instance, Spence and Robbins promoted the view that workaholism is a multidimensional concept that consists of work engagement, being work-driven, and work enjoyment [16]. Meanwhile, Scott tried to categorize workaholics as being compulsive-dependent, perfectionists, and achievement-oriented [17]. On the other hand, Ng, Sorensen, and Feldman defined workaholics as “those who enjoy the act of working, who are obsessed with working, and who devote long hours and personal time to work” by emphasizing the affective, cognitive, and behavioral aspects of workaholism [18]. However, there is criticism in considering affective aspects as a subdimension of workaholism because there is no consensus on reported affective experiences among workaholics [19,20]. Recently, Clark et al. newly conceptualized workaholism with four dimensions: addition to work, persistent thoughts about work, excessive physical or mental energy towards work, and detrimental effect on health and well-being [19,21]. Although various approaches and definitions of workaholism exist, all of them share two important characteristics: working excessively and working compulsively. Thus, in this study, we use the definition “the tendency to work excessively hard in a compulsive way” [20], which comprises of two subdimensions: working excessively and working compulsively.

Workaholism has been treated as a negative psychological state. For instance, workaholics are likely to work excessively beyond the demands of the organization [17,20]. They also tend to suffer from life problems due to excessively engaging themselves with work [17,22]. Workaholics are likely to ceaselessly think of their tasks and experience a feeling of guilt even after work [16].

On the other hand, work engagement has been regarded as a positive psychological and affective motivational state characterized by vigor, dedication, and absorption related to work [23,24]; vigor―having a high level of mental energy at work―drives people to invest their effort at work; dedication―being strongly engaged at work―leads people to experience a feeling of significance, pride, and inspiration at work; absorption―being fully engrossed and happily concentrated at work―makes it difficult for people to detach from their work and makes it feel like time passes by so fast. In contrast to workaholic behavior, engaged workers are not driven by an obsessive inner drive, but invest their time and efforts since they really enjoy their work [25]. To sum up, at the behavioral level, work engagement and workaholism can be expressed as “working very hard,” but at the cognitive level, they have different features. In this context, scholars have recently attempted to understand workaholism and work engagement through one unified perspective [8].

Snir and Harpaz introduced the concept of HWI—investing both time and effort in work—to unify the concepts of workaholism and work engagement. According to this theoretical perspective [10,26], workaholism and work engagement can also be viewed as negative and positive types of HWI, respectively. HWI research has focused on how workaholism and work engagement lead to different work-related outcomes such as well-being [25] and performance [27]. In order to understand how individuals develop negative or positive types of HWI, there is an increasing necessity to investigate various predictors. Studies on the antecedents of HWI have shown that both individual (internal) and situational (external) factors can predict workaholism and work engagement [8,9,28,29], but individual factors are often thought to be stronger predictors [8]. Recent research [8,26] supports the notion that personal characteristics (e.g., narcissism, passion for work) are stronger predictors of HWI, but studies on individual-level predictors are still lacking. Thus, at the individual level, the present study will consider work orientation (e.g., calling), attitude, and value toward one’s own work as predictors of HWI.

### 1.2. Calling and HWI

A calling refers to “a transcendent summons, experienced as originating beyond the self, to approach a particular life role in a manner oriented toward demonstrating or deriving a sense of purpose or meaningfulness, that holds other-oriented values and goals as primary sources of motivation” ([30], p. 427). This topic has attracted the attention of scholars from various psychological fields such as counseling, career development, and organizational and vocational psychology. Numerous studies have consistently shown that a calling is positively associated with work-related outcomes and individuals’ well-being [3,12,30,31]. For instance, those who view their work as a calling are likely to show greater organizational attachment and commitment [3,30,31]. However, some scholars argue that perceiving a calling might also harm workers [2,5]. Those who especially perceive a high level of calling tend to exhibit work engagement [4] and workaholic behaviors, such as working more and sacrificing personal time and effort [2,5,32], meaning that a calling may lead to the onset of HWI.

When it comes to linking a calling with HWI, it is relatively natural to accept the positive relationship between a calling and work engagement, since empirical research has consistently shown this relationship [4,33]. However, it might seem strange to associate the perception of a calling with workaholic behavior in a positive way since workaholics work excessively and obsessively and do not have a positive attachment to their work, which is opposite to the characteristics of workers who endorse their callings [30,34]. Interestingly, mixed results regarding the relationship between perceiving a calling and workaholism have been reported. Duffy and colleagues [6] reported no significant relationship between the two variables, while Lajom et al. [7] found a negative but nonsignificant relationship between them. In contrast, Hirschi and colleagues [35] found a small but positively significant relationship between calling and workaholism. We assumed that such mixed results might be due to the uncovered psychological mechanism underlying the relationship between perceiving a calling and work engagement or workaholism. Thus, our study utilizes the dualistic model of passion [13] as potential mediators in such a relationship in order to unpack the psychological mechanisms underlying it.

### 1.3. Mediating Role of Passion

Vallerand and colleagues [13] first introduced the dualistic model of passion (DMP) theory. According to the theory, passion, a “strong inclination toward a self-defining activity that people love, find important, and in which they invest time and energy” ([13], p. 98), consists of two types: harmonious (HP) and obsessive (OP) passions. HP is a more adaptive psychological process that leads people to freely participate and engage in their work; therefore, workers with HP tend to experience positive outcomes in terms of job creativity [36], work satisfaction [37], and good relationship with coworkers [38].

Meanwhile, OP is a less adaptive process, which includes uncontrolled pressure that drives people to engage in their work [39]. Although individuals with OP like the activities that they are passionate about, they tend to satisfy other psychological needs (e.g., self-esteem) from the activity, rather than being satisfied when engaging in the activity itself. Due to such a necessity to satisfy psychological needs, they tend to rely extremely on the activity as compensation. Those with OP are likely to experience burnout [37], work–family conflict [39], and psychological distress [13]. As the concept of HWI helps us understand work engagement and workaholism through a unified theoretical lens, Vallerand and colleagues’ [13] DMP theory explains HP and OP as positive and negative types of passion [13,40].

We attempted to reveal the psychological mechanisms underlying the relationship between perceiving a calling and HWI by using the dual-path mediation effects of passion (HP and OP). First, we posited that HP could mediate the positive relationship between perceiving a calling and work engagement. Individuals who view their work as a calling tend to regard their work as a central aspect of their life and love it [12]. HP manifests when someone engages in activities that they love [13]. Therefore, those with a calling can naturally foster HP [14], which results in active engagement in their work field.

Second, we also posited that OP could mediate a positive relationship between perceiving a calling and workaholism. According to previous research [7,41], OP and workaholism are closely related but are distinguished psychological constructs; they share core characteristics (e.g., being obsessed about work). The link between perceiving a calling and OP can be explained by the fact that individuals who endorse a calling consider their work as a core part of their identities [42]. When work conflates with identities, people begin to continuously use their personal resources in compulsive ways [35]. Additionally, when workers feel called to a certain work domain, obsessive thoughts about work are so powerful that they tend to dominate all other aspects of life [2,14]. Furthermore, workers with a calling tend to perceive work as a duty they should abide by [2]; such a feeling can force them to work obsessively at the expense of personal time and effort. In fact, previous empirical studies have supported the idea that individuals who view their work as a calling can compulsively work in order to live by their calling; they are more likely to work longer and are less likely to disengage from work [5,32]. For instance, in a study of zookeepers, those with a calling tended to sacrifice their personal time, income, and psychological comfort to carry out their work [2]. Collectively, we expect that perceiving a calling can influence both work engagement and workaholism through HP and OP.

### 1.4. Present Study and Hypotheses

**Figure 1:**
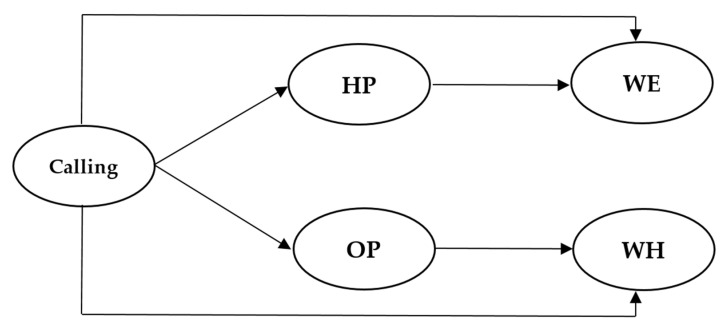
Hypothesized model. HP = harmonious passion; OP = obsessive passion; WE = work engagement; WH = workaholism.

In this study, we aim to reveal the influence of perceiving a calling on HWI (work engagement and workaholism), and the psychological mechanisms underlying such influence. We established a dual-path mediation model (Figure 1) to examine whether two types of passion (HP and OP) can explain how perceiving a calling influences work engagement and workaholism, respectively. Our hypotheses are as follows:

**Hypothesis 1a** **(H1a).** *Perceiving a calling is positively associated with work engagement.*

**Hypothesis 1b** **(H1b).** *Perceiving a calling is positively associated with workaholism.*

**Hypothesis 2a** **(H2a).** *Perceiving a calling is positively associated with harmonious passion.*

**Hypothesis 2b** **(H2b).** *Perceiving a calling is positively associated with obsessive passion.*

**Hypothesis 3a** **(H3a).** *Harmonious passion is positively associated with work engagement.*

**Hypothesis 3b** **(H3b).** *Obsessive passion is positively associated with workaholism.*

**Hypothesis 4a** **(H4a).** *Harmonious passion mediates the relationship between a calling and work engagement.*

**Hypothesis 4b** **(H4b).** *Obsessive passion mediates the relationship between a calling and workaholism.*

## 2. Methods

### 2.1. Sample

This study was approved by the University’s Institutional Review Board. American workers were randomly recruited using Amazon’s Mechanical Turk service. After completing a 10-min online survey, they received a certain amount of monetary reward. A total of 398 participants were recruited; all participants were over 18 years old, had worked at the same company for more than one year, were not self-employed or students, and had worked at least 20 h per week. The average age of the participants was 34.15 (SD = 9.46), and 59% of the respondents were male. Over half of the sample had a bachelor’s degree (54.8%) and 22. 9% had a high school diploma. The average tenure (i.e., working years in their current company) of participants was 6.88 years (SD = 6.04).

### 2.2. Measures

#### 2.2.1. Perceiving a Calling

The Calling and Vocation Questionnaire (CVQ) developed by Dik and colleagues [43] was used to assess the participants’ callings. The CVQ consists of three dimensions: transcendent summons, purposeful work, and prosocial orientation. Each dimension contains four items: transcendent summons (e.g., “I believe that I have been called to my current line of work”), purposeful work (e.g., “My work helps me live out my life’s purpose”), and prosocial orientation (e.g., “The most important aspect of my career is its role in helping to meet the needs of others”). All items are scored on a five-point scale ranging from 1 (not at all true of me) to 5 (absolutely true of me). The Cronbach’s alpha was 0.78 for transcendent summons, 0.93 for purposeful work, and 0.88 for prosocial orientation.

#### 2.2.2. Passion

The Passion Scale developed by Vallerand et al. [13] was used to assess the participants’ passions. It consists of two dimensions: HP and OP. Each dimension contains seven items: HP (e.g., “For me, it is passion that I still manage to control”) and OP (e.g., “I cannot imagine my life without [activity]”). All items were scored on a seven-point scale ranging from 1 (do not agree at all) to 7 (very strongly agree). The Cronbach’s alpha was 0.95 for HP and 0.95 for OP.

#### 2.2.3. Work Engagement

The nine-item Utrecht Work Engagement Scale (UWES) developed by Schaufeli [9] was used to assess the participants’ work engagement. Each dimension contained three items: vigor (e.g., “At my job, I feel strong and vigorous”), dedication (e.g., “I am enthusiastic about my job”), and absorption (e.g., “I am immersed in my work”), scored on a seven-point scale ranging from 0 (never) to 6 (everyday). The Cronbach’s alpha was 0.86 for vigor, 0.89 for dedication, and 0.84 for absorption.

#### 2.2.4. Workaholism

The Dutch Work Addiction Scale (DUWAS) developed by Schaufeli and colleagues [20] was used to assess the participants’ workaholic behaviors. It contains two dimensions: working excessively and working compulsively. Five items are used to assess working excessively (e.g., “I seem to be in a hurry and racing against the clock”), and five items to assess working compulsively (e.g., “It is important to me to work hard even when I don’t enjoy what I’m doing”). Each item is rated on a scale ranging from 1 (strongly disagree) to 5 (strongly agree). The Cronbach’s alpha equaled 0.70 for working excessively and 0.75 for working compulsively.

#### 2.2.5. Control Variables

In order to control for exogenous effects, demographic variables such as age, gender, educational level, and tenure were measured. Educational level was assessed using one item ranging 1 (high school diploma or less) to 4 (doctoral degree). Tenure was assessed with one question: “How long have you been working in your current company?”.

### 2.3. Data Analysis

First, descriptive statistics (means, standard deviations, correlations) were analyzed. Second, we used confirmatory factor analysis (CFA) in Mplus 6.0 to evaluate our measurement model. Next, model fit indices were utilized: chi-square, comparative fit index (CFI), Tucker–Lewis index (TLI), root mean square error of approximation (RMSEA), and standardized root mean square residual (SRMR). Based on Hu and Bentler’s [44] guidelines, values above 0.90 for CFI, above 0.90 for TLI, below 0.08 for RMSEA, and below 0.08 for SRMR were regarded as acceptable model fit. We performed a series of CFAs to confirm the discriminant validity of the constructs, and chi-square tests were used to compare different nested measurement models. Third, to test our hypotheses, we used structural equation modeling (SEM) with 95% bias-corrected confidence intervals (5000 bootstrapped samples; [45]). Lastly, a competitive model was discussed but not accepted.

## 3. Results

### 3.1. Preliminary Analysis

We conducted preliminary analyses using IBM SPSS 25.0. No univariate or multivariate outlier was found. Following the recommendations of Weston and Gore [46], we also examined the skewness and kurtosis of our data; no variables that approached the thresholds were detected (skewness > |3| or kurtosis > |10|).

### 3.2. Descriptive Statistics

Table 1 presents descriptive statistics for our study variables (means, standard deviations, and correlation coefficients). The results showed that all study variables were positively correlated with each other.

### 3.3. Measurement Model

CFA was conducted to examine the goodness of fit before testing our structural model and to test our measurement model based on five latent constructs (perceiving a calling, HP, OP, work engagement, and workaholism). The results showed that our measurement model (a five-factor model) fit the data well based on four of the five fit indices: $\chi^{2}$(199) = 555.763, *p* < 0.001, CFI = 0.95, TLI = 0.93, RMSEA = 0.08, and SRMR = 0.07. Compared to the fit of the model when all the variables were loaded on one latent factor ($\chi^{2}$(209) = 2398.346, *p* < 0.001, CFI = 0.68, TLI = 0.64, RMSEA = 0.18, and SRMR = 0.10), the fit of our measurement model was better.

### 3.4. Hypothesis Test

We established our hypothesized model―a partial dual mediation model―after controlling for age, gender, educational level, and tenure. We controlled for such variables by adding a path from control variables to mediators and dependent variables in our structural model. Our hypothesized model showed that perceiving a calling was significantly correlated with work engagement (*β* = 0.34, *p* < 0.01), but not with workaholism (*β* = 0.12, *p* = 0.41). Thus, Hypothesis 1a is supported, but Hypothesis 1b is not; that is, Hypothesis 1 is partially supported.

**Figure 2:**
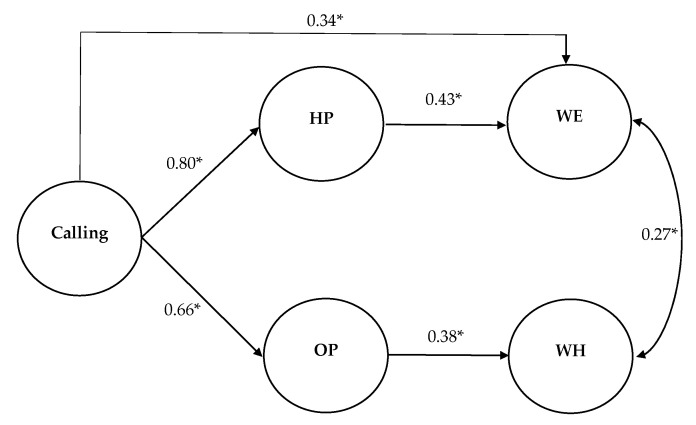
Structural model with standardized path estimate. Note: HP = harmonious passion; OP = obsessive passion; WE = work engagement; WH = workaholism; * *p* < 0.01. Control variables (age, gender, education level, and tenure) are not shown for simplicity.

Afterward, we checked the fit of a new partially mediating model, deleting the nonsignificant paths (calling→workaholism). The new model (Figure 2) showed a good fit ($\chi^{2}$(275) = 672.989, *p* < 0.001, RMSEA = 0.06, CFI = 0.94, TLI = 0.93, SRMR = 0.04), so we continued our hypothesis testing using this model, which is more parsimonious.

The results indicated that perceiving a calling was positively associated with HP (*β* = 0.80, *p* < 0.01) and OP (*β* = 0.66, *p* < 0.01). Thus, Hypotheses 2a and 2b are supported. Furthermore, our results also revealed a positive relationship between HP and work engagement (*β* = 0.43, *p* < 0.01) and OP and workaholism (*β* = 0.38, *p* < 0.01), supporting Hypotheses 3a and 3b. Lastly, perceiving a calling was both indirectly related to work engagement via HP (*indirect effect* = 0.34, *p* < 0.01, bootstrap 5000 samples, 95% CI: 0.17, 0.72) and to workaholism via OP (*indirect effect* = 0.25, *p* < 0.01, bootstrap 5000 samples, 95% CI: 0.03, 0.18), supporting Hypotheses 4a and 4b. Results of the indirect relations are presented in Table 2. Consequently, our results supported all hypotheses except H1b (Table 3).

### 3.5. Competitive Model

A competitive structural model can be considered since there might be criticism regarding significant and positive relationships between HP and workaholism (*r* = 0.13, *p* < 0.01) and obsessive HP and work engagement (*r* = 0.37, *p* < 0.01). Therefore, we established a competitive structural model that contains additional paths from HP to workaholism and OP to work engagement. Our competitive model also fitted well with our data ($\chi^{2}$(273) = 668.679, *p* < 0.001, CFI = 0.94, TLI = 0.93, RMSEA = 0.07, and SRMR = 0.05). However, it showed that HP was not significantly associated with workaholism (*β* = −0.18, *p* = 0.11) and OP was not significantly associated with work engagement (*β* = −0.04, *p* = 0.60). Therefore, we concluded that our research model was more parsimonious while supporting our hypothesized significant relationships.

## 4. Discussion

Our research contributes to the “work as a calling” theory (WCT) [1], which constitutes the first theoretical framework for callings, in that it empirically shows how perceiving a calling has double-edged sword effects. Even though WCT theoretically outlines the negative effects of perceiving a calling, empirical support has remained insufficient. Recent research [14] attempted to reveal the relationship between perceiving a calling and workaholism, but it was not enough to show the double-edged sword effect of perceiving a calling since work engagement was not taken into account in their model.

Drawing on the HWI [8] and DMP [13] theories, the present study presents its own unique findings regarding the association between perceiving a calling and two types of HWI (work engagement and workaholism) and the mediating roles of passion. Our results expand our knowledge on this topic by clarifying the mixed results about the relationship between perceiving a calling and workaholism and its positive and negative work-related outcomes. Our results also indicated that the relationship between perceiving a calling and workaholism was fully mediated by OP, while the relationship between perceiving a calling and work engagement was partially mediated by HP. That is, the type of passion (HP or OP) individuals develop towards their work impacts whether they experience positive (e.g., work engagement) or negative (e.g., workaholic behavior) work-related outcomes. Importantly, the results revealed that OP may be a key antecedent of work addiction behaviors, rather than perceiving a calling per se. Therefore, avoiding OP can help those with a strong calling to prevent the negative consequences of pursuing such calling.

Although our results showed that OP fully mediated the relationship between perceiving a calling and workaholism, a careful approach would be required to conclude that OP is fully responsible for the development of workaholic behaviors; the negative effects of a calling might have been overstated. As we have discussed, the direct effects of a calling on OP (*β* = 0.66, *p* < 0.01) and HP (*β* = 0.80, *p* < 0.01) were both positively strong, which implies that perceiving a calling is highly related to OP as well as HP. Even though the temporal precedence between perceiving a calling and developing passion needs to be investigated through longitudinal research, we carefully suggest that there are various mediators and moderators in the relationship between perceiving a calling and OP before making hasty generalizations.

The competitive model was established to investigate the possible impacts of HP on workaholism and OP on work engagement. This model was plausible due to its small but significant correlation results between HP and workaholism (*r* = 0.13, *p* < 0.01) and OP and work engagement (*r* = 0.37, *p* < 0.01). The results, however, showed that neither of them was significant, which means that HP and OP have their own distinct relationship with work engagement and workaholism, respectively. We assumed that the positive correlation between HP and workaholism occurred because of the overlapped portion (*r* = 0.61, *p* < 0.01) between HP and OP. Vallerand et al. [13], who are responsible for conceptualizing and developing HP and OP, reported that even though HP and OP are empirically distinct psychological concepts, they are highly correlated to each other (*r* = 0.46, *p* < 0.01) since they share high portions of passion. Thus, our results are aligned with their claim that the positive relationship between HP and OP is reasonable.

Stefano and Gaudiino [11] suggested the importance of understanding the subdimensions of workaholism and work engagement when conducting HWI research. They claimed that some of the subdimensions overlap between workaholism and work engagement: the positive relationship between working excessively and absorption (*g* = 0.34), working compulsively and absorption (*g* = 0.28), and working excessively and dedication (*g* = 0.14). Even though our research goal was not directly related to investigating the subdimensions of HWI, in order to clarify HP and OP’s unique effects on their subdimensions, we conducted additional analysis regarding the subdimensions of HWI. However, we used work engagement, vigor, dedication, and absorption as latent variables in our structural model. Additionally, workaholism, working excessively, and working compulsively were included as latent variables in our model. The results indicated that the model fit of the new structural model was not adequate and that HP was only positively associated with the subdimensions of work engagement and not with those of workaholism. Likewise, OP was only positively associated with the subdimensions of workaholism and not with those of work engagement. It means that even admitting the claim that some of the subdimensions of workaholism and work engagement might overlap [11], HP and OP play separate and important roles in predicting workaholism and work engagement.

This study also contributes to the literature in that we found perceiving a calling to be a new individual-level predictor of workaholism, which has been rarely investigated. Even though many scholars generally agree that individuals’ personalities cannot only be responsible for workaholism [47,48], studies on the antecedents of workaholism have been limited to the domain of personality. Our study, however, considers one’s calling―one’s form of work orientation―as a predictor of workaholism, which is novel. We found that not only individuals’ personalities but also perceiving a calling (attitude, belief, and cognitive processes associated with work) can directly or indirectly predict work engagement and workaholism. Specifically, our results (that perceiving a calling is highly related to OP but not directly related to workaholism) indicate the need to find which workers with a calling, and when, are more likely to develop OP and exhibit workaholic behaviors. We assumed dispositional factors that people already possess before searching for and perceiving a calling might influence the relationship between perceiving a calling and OP. For instance, among workers with a strong calling, those with perfectionism, neuroticism, narcissism, and conscientiousness tend to develop OP and exhibit workaholic behaviors [19,26,48]. That is, when those who possess such dispositional factors start to passionately pursue their calling, the possibilities of developing OP and workaholic behaviors might increase. Therefore, research regarding the interaction between calling and those personality traits would be required.

Organizational factors can also be considered as potential moderators that lead workers with a calling to or suppress them from developing OP and workaholic behaviors. Although the necessity of an integrative approach that includes individual-level and organizational-level predictors of workaholism has been raised [47,48], the studies on interaction effects have been limited. Workers with a calling, whose work experiences include organizations with competitive climates and high job demands, may boost compulsive attitudes and behaviors [5,9,20]. Conversely, organizational factors such as corporate social responsibility (CSR) and organizational support may suppress the development of workaholic behaviors. Previous research [49] has shown that CSR can be an effective strategy to boost employees’ meaning pursuits, which may increase their HP.

Lastly, the calling itself (e.g., unanswered calling) can result in OP and workaholic behaviors. Research on individuals perceiving and living by a calling, an unanswered calling, and no calling [50] revealed that those with an unanswered calling reported significantly lower physical and psychological health compared to the two other groups. According to DMP, people are more likely to develop OP when facing failure in fulfilling basic needs from activities that they are passionate about [13,51]. Likewise, individuals with an unanswered calling—those who cannot satisfy their basic psychological needs from the work they feel they are being called to—are more likely to compulsively invest an excessive amount of time and energy in work in order to compensate their deficiencies by finding meaning and living up to their calling.

Besides theoretical implications, our study has practical contributions for employers, human resources (HR) practitioners, and counselors. It may be granted that hard-working employees are preferred for employers to guarantee the sustainability of their business in a competitive society. In this regard, employers should pay more attention to the well-being of workers with a calling to prevent negative work-related outcomes such as workaholism, burnout, and turnover. In particular, employers are recommended to employ HR practices to help employees develop HP rather than OP. Supportive and autonomous HR practices [52] (e.g., helping employees to experience different work roles through job rotation) can give enough opportunities for employees to freely choose their work activities, which is an essential prerequisite for developing HP. These practices also help employees satisfy their basic psychologic needs, preventing the development of OP. Furthermore, career counselors or corporate counselors can incorporate our findings into their treatments or interventions. For the clients with no calling, counselors can help them find and develop their calling by realizing their vocational identity and their life purpose or meaning. For those with a calling, counselors should prioritize identifying whether their calling is compulsively intensified in their work domain. If so, counselors are recommended to intervene to satisfy their basic psychological needs from various life and work domains in order to prevent them from compulsively engaging in work as a compensative measure.

Our results also contribute to the understanding of future society. Researchers and corporate managers should pay attention to the millennials’ unique characteristics (born from 1981 to 2000; [53]) since they will soon become the leaders of the future era. For instance, millennials tend to show delicate sensitivity toward an organization’s ethical issues [54,55], seek self-actualization and accomplishment, and believe they can obtain those from their job [56]. This generation also values meaningful and satisfying work [57,58], which implies that they are more likely to have interests in searching, perceiving, and living by their calling. As the need for caring about millennials’ callings increases [59], an accurate understanding of the negative effects of perceiving a calling is important to prevent negative outcomes. In this perspective, we expect that the understanding of the double-edged effects of perceiving a calling and its psychological mechanisms will help researchers and corporate managers prepare to help millennials live by their calling while preventing negative outcomes.

### Limitations and Future Studies

Despite its contributions, our study has several limitations. First, our study was cross-sectional, meaning that causal inferences cannot be inferred. Even though we posited that perceiving a calling would predict passion based on previous research, further longitudinal research will be required to determine causality. Second, as discussed in the previous section, the interaction effects of various dispositional (e.g., narcissism, low self-esteem, perfectionism) and situational (competitive climate, CSR) factors need to be clarified to better understand the psychological mechanisms and limits of a calling’s effects on HWI. Third, the sample of this study was limited to American workers. There might be differences in understanding and endorsing passion according to culture. For instance, in South Korea, where achieving success has been extremely emphasized, OP is defined as being painfully but ceaselessly clinging to one domain [60]. Such a painful passion is often regarded as a virtue in successful Koreans. Therefore, it is possible that due to cultural aspects, the link between OP and workaholism can weaken or disappear.

## 5. Conclusions

We have attempted to broaden the literature on calling, passion, and HWI among American workers. Based on the HWI theoretical framework, our study at least partially reveals how perceiving a calling affects both workaholism and work engagement. Furthermore, by utilizing DMP, our study investigated the psychological mechanisms underlying the positive effects of perceiving a calling on work engagement and workaholism. Our results show that HP fully mediates the association between perceiving a calling and work engagement, while OP partially mediates the association between perceiving a calling and workaholism. This study contributes to the extant literature by clarifying how perceiving a calling affects work engagement and workaholism differently through two kinds of passion. This study has several practical implications as well. Employers should treat workers with a calling carefully to avoid the development of OP or workaholic behaviors by providing supportive HR practices. Furthermore, counselors should guide their clients with a precise diagnosis of their calling and passion and provide adequate intervention in order to deter them from experiencing negative psychological outcomes. Lastly, longitudinal and cross-cultural studies are required to be able to generalize our results.

## Figures and Tables

**Table 1:** Descriptive statistics.

Variables	M	SD	1	2	3	4	5	6	7
1. Age	35.19	9.46							
2. Education Level	2.07	0.86	0.07						
3. Tenure	6.88	6.04	0.41 **	−0.03					
4. Calling	3.18	1.05	−0.03	0.08	−0.09				
5. Harmonious Passion	4.62	1.52	−0.01	0.03	−0.20	0.71 **			
6. Obsessive Passion	3.40	1.69	−0.13 **	−0.01	−0.05	0.54 **	0.61 **		
7. Work Engagement	5.10	1.35	0.09	0.06	0.01	0.60 **	0.64 **	0.37 **	
8. Workaholism	3.29	0.74	0.02	−0.05	−0.03	0.11 *	0.13 **	0.25 **	0.24 **

*N* = 398; * *p* < 0.05, ** *p* < 0.01.

**Table 2:** Indirect relations for the structural model.

Paths	Standardized Indirect Effect		Bootstrap Bias Corrected 95% Confidence Interval	
	β	*SE*	Lower bound	Upper bound
Calling→HP→WE	0.34	0.10	0.17	0.72
Calling→OP→WH	0.25	0.06	0.03	0.18

HP = harmonious passion; OP = obsessive passion; WE = work engagement; WH = workaholism.

**Table 3:** Hypothesis testing from the structural model.

Hypothesized Path	Support?
1a. Calling→Work Engagement	Supported
1b. Calling→Workaholism	Not supported
2a. Calling→Harmonious Passion	Supported
2b. Calling→Obsessive Passion	Supported
3a. Harmonious Passion→Work Engagement	Supported
3b. Obsessive Passion→Workaholism	Supported
4a. Calling→Harmonious Passion→Work Engagement	Supported
4b. Calling→Obsessive Passion→Workaholism	Supported

## References

[1] Duffy R.D., Dik B.J., Douglass R.P., England J.W., Velez B.L. (2018). Work as a calling: A theoretical model. J. Couns. Psychol..

[2] Bunderson J.S., Thompson J.A. (2009). The call of the wild: Zookeepers, callings, and the double-edged sword of deeply meaningful work. Admin. Sci. Quart..

[3] Cardador M.T., Dane E., Pratt M.G. (2011). Linking calling orientations to organizational attachment via organizational instrumentality. J. Vocat. Behav..

[4] Hirschi A. (2012). Callings and work engagement: Moderated mediation model of work meaningfulness, occupational identity, and occupational self-efficacy. J. Couns. Psychol..

[5] Keller A.C., Spurk D., Baumeler F., Hirschi A. (2016). Competitive climate and workaholism: Negative sides of future orientation and calling. Personal. Individ. Differ..

[6] Duffy R.D., Douglass R.P., Autin K.L., England J., Dik B.J. (2016). Does the dark side of a calling exist? Examining potential negative effects. J. Posit. Psychol..

[7] Lajom J.A.L., Amarnani R.K., Restubog S.L.D., Bordia P., Tang R.L. (2018). Dualistic passion for work and its impact on career outcomes: Scale validation and nomological network. J. Career Assess..

[8] Snir R., Harpaz I. (2012). Beyond workaholism: Towards a general model of heavy work investment. Hum. Resour. Manag. Rev..

[9] Schaufeli W.B. (2016). Heavy work investment, personality and organizational climate. J. Manag. Psychol..

[10] Taris T., van Beek I., Schaufeli W.B., Harpaz I., Snir R. (2015). The beauty versus the beast: On the motives of engaged and workaholic employees. Heavy Work Investment: Its Nature, Sources, Outcomes and Future Directions.

[11] Di Stefano G., Gaudiino M. (2019). Workaholism and work engagement: How are they similar? How are they different? A systematic review and meta-analysis. Eur. J. Work Organ. Psychol..

[12] Wrzesniewski A., McCauley C., Rozin P., Schwartz B. (1997). Jobs, careers, and callings: People’s relations to their work. J. Res. Personal..

[13] Vallerand R.J., Houlfort N., Fores J., Gilliland S.W., Steiner D.D., Skarlicki D.P. (2003). Passion at work: Toward a new conceptualization. Emerging Perspectives on Values in Organizations.

[14] Dalla Rosa A., Vianello M. (2020). Linking calling with workaholism: Examining obsessive and harmonious passion as mediators and moderators. J. Career Assess..

[15] Oates W. (1971). Confessions of a Workaholic: The Facts about Work Addiction.

[16] Spence J.T., Robbins A.S. (1992). Workaholism: Definition, measurement, and preliminary results. J. Personal. Assess..

[17] Scott K.S., Moore K.S., Miceli M.P. (1997). An exploration of the meaning and consequences of workaholism. Hum. Relat..

[18] Ng T.W., Sorensen K.L., Feldman D.C. (2007). Dimensions, antecedents, and consequences of workaholism: A conceptual integration and extension. J. Organ. Behav. Int. J. Ind. Occup. Organ. Psychol. Behav..

[19] Clark M.A., Michel J.S., Zhdanova L., Pui S.Y., Baltes B.B. (2016). All work and no play? A meta-analytic examination of the correlates and outcomes of workaholism. J. Manag..

[20] Schaufeli W.B., Taris T.W., Bakker A.B. (2008). It takes two to tango: Workaholism is working excessively and working compulsively. The Long Work Hours Culture: Causes, Consequences and Choices.

[21] Clark M.A., Smith R.W., Haynes N.J. (2020). The Multidimensional Workaholism Scale: Linking the conceptualization and measurement of workaholism. J. Appl. Psychol..

[22] Cherrington D.J. (1980). The Work Ethic.

[23] Macey W.H., Schneider B. (2008). The meaning of employee engagement. Ind. Organ. Psychol..

[24] Schaufeli W.B., Salanova M., González-Romá V., Bakker A.B. (2002). The measurement of engagement and burnout: A two sample confirmatory factor analytic approach. J. Happiness Stud..

[25] Shimazu A., Schaufeli W.B. (2009). Is workaholism good or bad for employee well-being? The distinctiveness of workaholism and work engagement among Japanese employees. Ind. Health.

[26] Falco A., Girardi D., Di Sipio A., Calvo V., Marogna C., Snir R. (2020). Is narcissism associated with heavy work investment? The moderating role of workload in the relationship between narcissism, workaholism, and work engagement. Int. J. Environ. Res. Public Health.

[27] Shimazu A., Schaufeli W.B., Kamiyama K., Kawakami N. (2015). Workaholism vs. work engagement: The two different predictors of future well-being and performance. Int. J. Behav. Med..

[28] Fleck S., Inceoglu I. (2010). A comprehensive framework for understanding and predicting engagement. The Handbook of Employee Engagement: Perspectives, Issues, Research, & Practice.

[29] Liang Y.W., Chu C.M. (2009). Personality traits and personal and organizational inducements: Antecedents of workaholism. Soc. Behav. Pers. Int. J..

[30] Dik B.J., Duffy R.D. (2009). Calling and vocation at work: Definitions and prospects for research and practice. Couns. Psychol..

[31] Duffy R.D., Dik B.J., Steger M.F. (2011). Calling and work-related outcomes: Career commitment as a mediator. J. Vocat. Behav..

[32] Clinton M.E., Conway N., Sturges J. (2017). “It’s tough hanging-up a call”: The relationships between calling and work hours, psychological detachment, sleep quality, and morning vigor. J. Occup. Health Psychol..

[33] Xie B., Xia M., Xin X., Zhou W. (2016). Linking calling to work engagement and subjective career success: The perspective of career construction theory. J. Vocat. Behav..

[34] Graves L.M., Ruderman M.N., Ohlott P.J., Weber T.J. (2012). Driven to work and enjoyment of work: Effects on managers’ outcomes. J. Manag..

[35] Hirschi A., Keller A.C., Spurk D. (2019). Calling as a double-edged sword for work-nonwork enrichment and conflict among older workers. J. Vocat. Behav..

[36] Liu D., Chen X.P., Yao X. (2011). From autonomy to creativity: A multilevel investigation of the mediating role of harmonious passion. J. Appl. Psychol..

[37] Carbonneau N., Vallerand R.J., Fernet C., Guay F. (2008). The role of passion for teaching in intrapersonal and interpersonal outcomes. J. Educ. Psychol..

[38] Philippe F.L., Vallerand R.J., Houlfort N., Lavigne G.L., Donahue E.G. (2010). Passion for an activity and quality of interpersonal relationships: The mediating role of emotions. J. Pers. Soc. Psychol..

[39] Vallerand R.J. (2010). On passion for life activities: The dualistic model of passion. Advances in Experimental Social Psychology.

[40] Gorgievski-Duijvesteijn M., Bakker A., Albrecht S.L. (2010). Passion for work: Work engagement versus workaholism. New Horizons in Management. Handbook of Employee Engagement: Perspectives, Issues, Research and Practice.

[41] Birkeland I.K., Buch R. (2015). The dualistic model of passion for work: Discriminate and predictive validity with work engagement and workaholism. Motiv. Emot..

[42] Elangovan A.R., Pinder C.C., McLean M. (2010). Callings and organizational behavior. J. Vocat. Behav..

[43] Dik B.J., Eldridge B.M., Steger M.F., Duffy R.D. (2012). Development and validation of the calling and vocation questionnaire (CVQ) and brief calling scale (BCS). J. Career Assess..

[44] Hu L.T., Bentler P.M. (1999). Cutoff criteria for fit indexes in covariance structure analysis: Conventional criteria versus new alternatives. Struct. Equ. Model. Multidiscip. J..

[45] Nevitt J., Hancock G.R. (2001). Performance of bootstrapping approaches to model test statistics and parameter standard error estimation in structural equation modeling. Struct. Equ. Model..

[46] Weston R., Gore P.A. (2006). A brief guide to structural equation modeling. Couns. Psychol..

[47] Atroszko P.A., Demetrovics Z., Griffiths M.D. (2019). Beyond the myths about work addiction: Toward a consensus on definition and trajectories for future studies on problematic overworking: A response to the commentaries on: Ten myths about work addiction (Griffiths et al., 2018). J. Behav. Addict..

[48] Griffiths M.D., Demetrovics Z., Atroszko P.A. (2018). Ten myths about work addiction. J. Behav. Addict..

[49] Choi J., Sohn Y.W., Lee S. (2020). The effect of corporate social responsibility on employees’ organizational citizenship behavior: A moderated mediation model of grit and meaning orientation. Sustainability.

[50] Gazica M.W., Spector P.E. (2015). A comparison of individuals with unanswered callings to those with no calling at all. J. Vocat. Behav..

[51] Ryan R.M., Deci E.L. (2000). Self-determination theory and the facilitation of intrinsic motivation, social development, and well-being. Am. Psychol..

[52] Dalla Rosa A., Vianello M., Anselmi P. (2019). Longitudinal predictors of the development of a calling: New evidence for the a posteriori hypothesis. J. Vocat. Behav..

[53] Gursoy D., Chi C.G.Q., Karadag E. (2013). Generational differences in work values and attitudes among frontline and service contact employees. Int. J. Hosp. Manag..

[54] Klimkiewicz K., Oltra V. (2017). Does CSR enhance employer attractiveness? The role of millennial job seekers’ attitudes. Corp. Soc. Responsib. Environ. Manag..

[55] Connell J.A., McMinn N.E., Bell N. (2012). How will the next generation change the business world? A report on a survey. Insights Chang. World J..

[56] Guillemette M. (2009). And now a word from the millennials (as interpreted by a boomer). CPA Prac. Manag. Forum.

[57] Hirschman C. (2006). Here they come. Hum. Resour. Exec..

[58] Wey Smola K., Sutton C.D. (2002). Generational differences: Revisiting generational work values for the new millennium. J. Organ. Behav. Int. J. Ind. Occup. Organ. Psychol. Behav..

[59] Hammer E.E. (2015). Shifts in calling: An emphasis on calling for millennials. Am. J. Manag..

[60] Hong M.S., Jung Y.S., Sohn Y.W. (2016). Validation of the Korean passion scale. Korean J. Soc. Pers. Psychol..

